# Deciphering the Cape Gooseberry Fruits Mycobiome for Further Safety Improvement Postharvest

**DOI:** 10.3390/foods13203248

**Published:** 2024-10-12

**Authors:** Gabriela N. Tenea, Diana Molina

**Affiliations:** Biofood and Nutraceutics Research and Development Group, Faculty of Engineering in Agricultural and Environmental Sciences, Universidad Técnica del Norte, Ibarra 100150, Ecuador

**Keywords:** *Botrytis caroliniana*, *Candida railenensis*, cape gooseberry, ITS2 metabarcoding, *Meyerozyma guilliermondii*, organic management system

## Abstract

Cape gooseberries are exquisitely flavored fruits; their rapid deterioration reduces their shelf life. Understanding the unique mycobiome of fruit peels is an essential step in identifying the taxa causing postharvest loss. The current study proposes to analyze the fungal communities of cape gooseberry peels collected from an organic orchard at unripe and ripe stages and purchased from open-air market sites, using the ITS2 region metabarcoding. According to the Kruskal–Wallis test, there were no statistically significant differences found in either the phylogenetic or non-phylogenetic alpha diversity indices. Significant differences in fungal communities were observed between the market and orchard groups based on beta diversity results. *Ascomycota* (85.72–96.76%), *Basidiomycota* (3.21–13.91%), and *Chytridiomycota* (0.07–9.35%) were the most common fungal phyla, their abundance varying with the ripening stage and origin. *Dothideomycetes* in the orchard group and *Saccharomycetes* in the market group were the two most prevalent classes. Furthermore, we investigate which taxa showed a significant difference in abundance between the two conditions (market vs. orchard) using the analysis of compositions of microbiomes with bias correction (ANCOM-BC) test. Regardless of the phase, the orchard samples exhibited a notable increase in the mean absolute abundance of various beneficial fungal taxa, including *Tilletiopsis washingtonensis* and *Articulospora proliferata*, whereas the market samples demonstrated a high abundance of harmful yeasts and molds such as *Meyerozyma guilliermondii, Candida railenensis*, and *Botrytis caroliniana*. Although it is unclear how these microorganisms augment at the market sites and might impact the fruit quality after harvest, from a fruit safety perspective, it is essential to comprehend the diversity and variation of the mycobiome composition at different ripening stages to further develop strategies to improve food safety postharvest.

## 1. Introduction

Cape gooseberry (*Physalis peruviana* L.) locally referred as uvilla or uchuva is a perennial plant of the *Solanaceae* family [1]. The fruit is rich in antioxidants, has a delicious flavor, and includes soluble pectin, vitamins, phenolic compounds, and a variety of active molecules [2,3]. In Northern Ecuador, this crop is grown at 2000 and 3000 m above sea level, where temperatures are typically moderately cold, by some small-scale farmers and the indigenous community as a valuable daily source of income [4]. Yields from 104 to 350 tons/ha; according to AGROCALIDAD, 40% of the total production of organic cape gooseberries is cultivated in the Imbabura province [4]. Because of stringent quality standards that reject products with flaws, this fruit is frequently wasted [5]. Furthermore, the fruit market’s potential is restricted by the quick deterioration that occurs after harvest [6].

Fruits are particularly vulnerable to microbial deterioration because of differences in their chemical makeup, including their pH level and moisture content [7]. In addition, improper environmental conditions during harvesting, transit, storage, and marketing, as well as the activity of their enzymes, may favor spoiling [7]. A considerable reduction in shelf life and quality harm is caused by both physiological and compositional changes postharvest [8]. According to an early report, *Alternaria alternata* and *Botrytis cinerea* were detected in cape gooseberry plants [9]. Moreover, based on morphological characterization and through rDNA sequencing of the ITS region, 75 ascomycetous were identified in unhealthy necrotic and straw-colored plant leaves of cape gooseberry obtained from the Pichincha Province of Ecuador [1]. *Alternaria* was the most prevalent genus followed by several other fungal taxa such as *Epicoccum, Diaporthe*, and *Xylaria.*

In Ecuador, inexpensive retail establishments in the local market are often devoid of basic facilities, and produce is improperly stored, compromising the sites’ hygiene standards. Recently, we reported the first study on cape gooseberry bacteriome [10]. The phytopathogen *Candidatus Liberibacter* was abundant in the fruits collected from the field, while fruits from the market showed a high content of animal-origin pathogens [10]. Furthermore, some fruits that were bought from the market also contained a pathogenic antibiotic resistant *Escherichia coli*, which suggests a possible cross contamination at the market sites [11]. 

Nonetheless, not much is known about the composition and mycobiome diversity linked to cape gooseberry fruits. These microorganisms, such as endophytes and epiphytes, inhabit vast environments and play an important role in agroecosystem biodiversity or can be detrimental to fruits if they are pathogenic [12,13]. Like bacteriome contamination, fungal colonization on fruit surfaces after harvesting is a serious issue. The possibility of human contamination of the samples collected from the orchard was not ruled out, as the fruit’s calyx is manually removed before being sold in the market. In addition, the vendors place the fruits in plastic boxes without using gloves or any other instrument. Nonetheless, there are at least two stages in the selection process where workers handle the fruits, decreasing their safety. Furthermore, it takes roughly two days from harvesting to the selling locations. However, once at the open-air market, the fruits are kept at room temperature for a few days if they are not sold that day, which allows both bacteria and fungi to grow or remain on the surface.

Due to the lack of information regarding the mycobiome in cape gooseberries at the transition from unripe to ripe stage, and the high demand for the consumption of this fruit, we used an ITS2-based metagenomic approach in the current study to decode the fungal composition of fruits harvested from a local organic certified orchard at ripe phase two: green, and phase four: yellow, and the fruits obtained from different open-air market sites (ready-to-eat fruits). The fungus mycobiome associated with fruits offers a way to increase fruit safety and support the development of extra precautionary measures against postharvest contamination. 

## 2. Materials and Methods

### 2.1. Fruit Sampling and Processing

Fruits were collected once without seasonal variation as a parameter from an organic certified producer (Imbabura Province, Parroquia San José de Quirquinche, 0°18′00″ N 78°16′00″ O) on May 2023. The calyx color was the ripe stage indicator [10]. Phase two is with green calyx and green fruit, and phase four is with straw-colored calyx and yellow fruit as shown in Figure S1. Fruits were randomly collected from the orchard setting according to the following design: 15 fruits (≈50 g) × field row × 6 field rows × 2 ripeness stages × 3 repetitions (# 540 fruits). Fruits (ripe four to five: ready-to-eat without calyx) were also bought from Ibarra city’s retail market (Imbabura Province, 0°21′46′′ N 78°07′48′′ O) as follows: 15 fruits × 6 sites × 3 repetitions (# 270 fruits). After carefully removing each fruit’s exocarp with a sterile knife, these were submerged in liquid nitrogen; the mixture was homogenized by grinding it fine in a mortar and pestle that had been chilled beforehand. DNA miniprep kit (ZymoBIOMICS, # D4304, Ecogen SL, Barcelona, Spain) was used for genomic DNA isolation [14].

### 2.2. Library Construction, Sequencing, Data Processing, and Analysis

An Illumina Novaseq platform (paired-end 150 bp reads, Illumina, San Diego, CA, USA) was used for sequencing [15]. The universal fungal primers ITS 86F (5′-GTGAATCATCGAATCTTTGAA-3′) and ITS 4 (5′-TCCTCCGCTTATTGATATGC-3′) for the amplification of ITS2 region were used [16]. The KAPA HiFi HotStart ReadyMix (# 2GFHSRMKB, Sigma Aldrich, St. Louis, MO, USA) was used in all polymerase chain reactions. To eliminate free primers and primer dimers from the amplicons, magnetic beads were used in a washing step. Up to 96 libraries were pooled using all the Nextera XT indices in preparation for sequencing. The purification, sequencing, and library preparation procedures were carried out as described [15]. Taxonomic classification of FASTq files was checked through a quality and filtering process. Data from microbiome sequencing based on marker genes was analyzed using the QIIME v.2 (Quantitative Insights into Microbial Ecology) pipeline [17], version 2023.5. After denoising the sequences with the denoise wrapper, the ITS2 region was extracted [18]. DADA2 was used for denoising and clustering [19]. DADA2 generates higher-resolution tables of amplicon sequence variables (ASVs) [20]. USEARCH 6.1 was used to find and filter chimera sequences [21]. The ASVs were also compared to a reference database of sequences whose taxonomic composition was established. Removal was carried out from sequences related to eukaryotes, mitochondria, and chloroplasts. The database used is the UNITE Fungal ITS Database 9.0, based on a set of FASTA files from UNITE Community [22]. There were two types of negative controls used: (1) every step of the DNA extraction process, as well as the ones that followed, included a blank extraction control. This empty control did not have any input data. (2) A DNA-free water as the input for library formation and subsequent sequencing was used as control [15].

### 2.3. Rarefaction Curves

Rarefaction curves are used to assess a sample’s biological diversity or characteristics and how it varies based on the number of sequences (reads) analyzed [23]. Examining how the number of new species (or features) detected rises as more reads are sequenced is the primary objective of rarefaction curves.

### 2.4. Alpha and Beta Diversity Analysis

Alpha and beta diversity were examined as previously mentioned [10]. The metrics listed below were determined: Faith’s phylogenetic diversity as a qualitative indicator of community richness that considers the phylogenetic relationships between traits [24]; the Shannon diversity index that quantify species diversity within groups and evenness/Pielou uniformity which measures variety in addition to species richness; and observed features, which is a qualitative indicator of community wealth [24]. Boxplot figures were used to display the results. To ascertain whether the samples were taken from the same distribution, the alpha diversity data were compared using a one-way ANOVA on ranks or a nonparametric Kruskal–Wallis test. For beta diversity, two methods were used: phylogenetic (UniFrac distance) and nonphylogenetic (Bray–Curtis dissimilarity) [25].

Using weighted and unweighted UniFrac distance matrices and 999 Monte Carlo permutations, the statistical significance of the observed differences was assessed. Using QIIME 2.0, principal coordinates analysis (PCoA) was used to correlate the composition of the fungal community with the sample group.

### 2.5. Statistical Significance Tests

The analysis of similarities (ANOSIM) was used to investigate the possibility that two or more groups of samples were the same [10]. The R test statistic was used to see if there were any differences between the groups under the null hypothesis [26]. Furthermore, the taxa with significantly differential abundances were found using the ANCOM-BC analysis [27]. ANCOM-BC estimates unknown sampling fractions, corrects the bias induced by their differences through a log-linear regression model that includes the estimated sampling fraction as offset terms and identifies differentially abundant taxa based on the variable of interest. ANCOM-BC is calculated based on a reference group. This is necessary to interpret differences in the abundance of features (such as taxa) between different groups or conditions. The resulting graph shows the differential abundance of features (taxa) between two or more groups of interest relative to the reference group. To ascertain the similarities or differences between the groups at the genus level, heatmaps and hierarchical clustering using the unweighted pair group method (UPGMA with Euclidean distance) were employed.

## 3. Results and Discussion

### 3.1. Fungal Alpha-Diversity in Cape Gooseberry Fruits

A total of 4,878,808 filtered reads were obtained from the amplicon sequencing after quality assessment (Table S1). Moreover, after filtration to remove non-target reads (like mitochondria or chloroplasts), sequences were classified as amplicon sequence variants (ASVs) (Table 1). Based on the rarefaction curve analysis, most of the taxa in the samples were sequenced at a depth of 150,000 (Figure S2). The curve’s tendency towards the plateau level has led to the identification of most of the species (or characteristics) in the sample. The alpha diversity analysis indicates that there is comparable fungal diversity among fruits originating from both orchard and market sites (Table 1).

**Figure 1:**
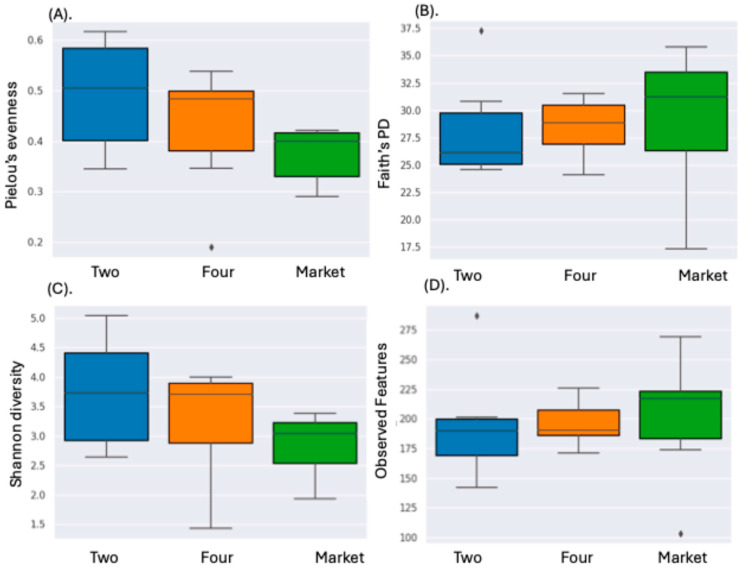
Box and whisker plots of alpha diversity values of fungal communities analyzed. (**A**) Pielou’s evenness; (**B**) Faith PD (phylogenetic diversity); (**C**) Shannon diversity index; (**D**) observed features. Legend: Two: fruits collected from the orchard phase two; Four: fruits collected from the orchard phase four; Market: fruits purchased from the market sites. Each box’s middle line aligns with the mean, and its upper and lower bounds are spaced one standard deviation apart from the mean. The highest and lowest values of each group are reached by the whiskers that extend above and below the box. The significance was determined Kruskal–Wallis; the values were considered significant when *p* < 0.05.

The unripe fruits (phase two) from the orchard presented the highest level of fungal diversity (average Shannon indices of 3.73), which was in line with the ASV data and the Shannon diversity index. The market fruits had the lowest Shannon index of 2.84 (Table 1). No statistically significant differences between the groups (two vs. market; four vs. market) for either the non-phylogenetic or phylogenetic alpha diversity indices were revealed by Kruskal–Wallis test (Figure 1A–D; Table S2). Comparably, when we collected cape gooseberries from the orchard and the market, we did not find any appreciable variations in the bacterial alpha diversity [10]. Furthermore, based on a conventional bacteriological technique used on cape gooseberry fruits bought from the market, an extensive number of molds and yeasts were detected [28].

### 3.2. Fungal Beta-Diversity in Cape Gooseberries

**Figure 2:**
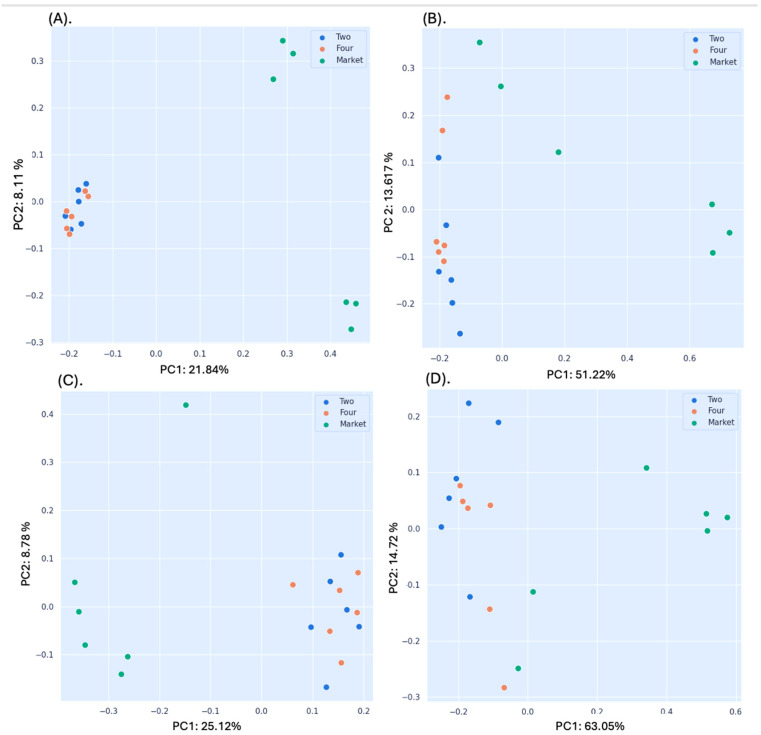
Principal coordinate analysis (PCoA) plots. (**A**) Bray–Curtis dissimilarity indices; (**B**) Jaccard distance; (**C**) unweighted UniFrac distance; (**D**) weighted UniFrac distance. Statistics were calculated using pairwise PERMANOVA with 999 permutations. Legend: Two: fruits collected from the orchard phase two; Four: fruits collected from the orchard phase four; Market: fruits purchased from the market sites.

Significant differences between the orchard phase four and market groups were found based on the non-phylogenetic distance results conducted by Jaccard (pseudo-F = 3.567, *p* = 0.002; PERMANOVA) and Bray–Curtis analyses (pseudo-F = 5.466, *p* = 0.006; PER-MANOVA) (Figure 2A,B; Table 2). Similarly, fruits at phase two and from the market showed a statistically significant difference using both the Bray–Curtis and Jaccard non-phylogenetic indexes (Table 2). Furthermore, significant differences between the market and orchard were identified through the application of the unweighted UniFrac distance as a phylogenetic index (Table 2). Regardless of their level of ripening, the market group samples separated from the orchard samples, as shown in the PCoA map for the abundance unweighted UniFrac distance (Figure 2C). Significant variations in beta diversity were discovered based on the weighted UniFrac distance (Figure 2D; Table 2). Loading the market samples in a positive direction yielded 63.05% of the total variance explained by variable F1 (Axis 1), while loading the orchard settings (phases two and four) yielded 14.72% of the variance. The Jaccard (pseudo-F = 1.181; *p* = 0.009; PERMANOVA) and Bray–Curtis non-phylogenetic distances showed significant differences between phases two and four of the orchard sample, but not in the latter (Table 2).

Furthermore, no statistical significance was found in the phylogenetic index unweighted and weighted UniFrac distances. Additionally, group four and the market had R values of almost 1.0 (0.57 and 0.60) for both group two and the market, indicating that there was dissimilarity between the groups according to the results of the Bray–Curtis similarity analysis (ANOSIM). Similarly, groups two and four that originated from the orchard had a uniform distribution (R-value of 0.032). These results supported the earlier profile of the cape gooseberry bacterial community, which demonstrated that fruits bought from the market have a different microbiome than fruits grown in the field [10]. Considering that the fruits were obtained from an organic orchard where no fungicidal treatments was applied, the susceptibility to contamination postharvest may increase. In addition, farmers gather cape gooseberry fruits with calix, which may, to a lesser extent, contain plant pathogens that can spread to the fruit after the calix is removed by hand. Thus, a substantial reduction in fruit quality and a decrease in shelf life might be attributed to the microbiome composition changing postharvest.

### 3.3. Cape Gooseberry Fungal Communities Taxonomic Assignment at Unripe and Ripe Stages

**Figure 3:**
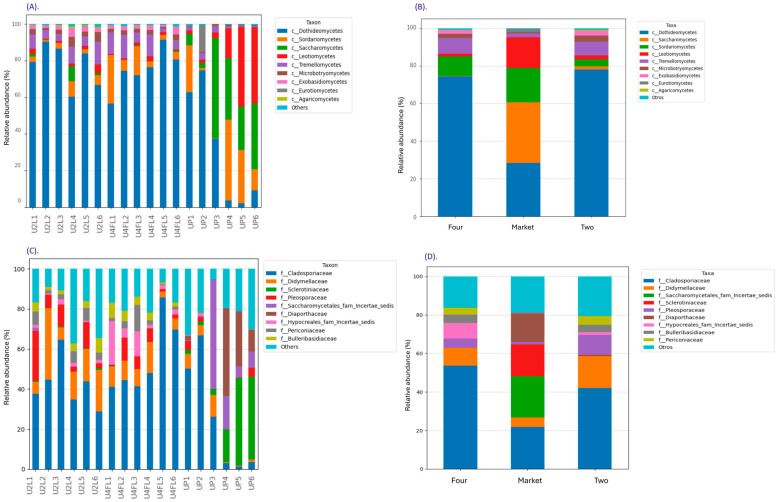
Fungal relative abundance (%) at the class and family levels across the samples (**A**,**C**) and groups (**B**,**D**). The stacked bar plots were constructed based on the relative abundance of the top 10 fungal classes/families, while “Other” category was defined as the sum of all classifications with less than 0.50% abundance. Legend: U2L1–U2L6: fruits collected from the organic orchard ripe phase two; U4FL1–U4FL6: fruits collected from the organic orchard ripe phase four; UP1–UP6: fruits purchased from the market sites.

The most common phyla found in the different fruits after harvest are *Ascomycota* and *Basidiomycota;* the phyla abundance varies with the host genotype, ripe stage, environmental factors, and season [29]. In the current study, six phyla and 29 classes were determined by the taxonomic assignment of ASVs. *Ascomycota* (varying from 85.72 to 95.76%) and *Basidiomycota* (varying from 3.21 to 13.91%) were the most abundant fungal phyla among the groups, followed in very low abundance by *Chytridiomycota, Mucoromycota, Mortierellomycota*, and Fungi-phy-Incertae-sedis (0.45–2.89%) (Table S3). Moreover, *Saccharomycetes, Sordariomycetes, Leotiomycetes*, and *Dothideomycetes* were the most common classes in the market samples, whereas *Tremellomycetes* were the most common classes detected in the orchard (Figure 3A,B). Recent mycobiome study performed on apple peel showed that *Ascomycota* (79.8%) and *Basidiomycota* (9.3%) dominated the fungal community, while other phyla, including *Entomophthoromycota, Mortierellomycota, Chytridiomycota,* and *Mucoromycota*, were also found, but at a lower relative abundance [30]. *Dothideomycetes* were detected in several tropical fruit peel (banana, guava, mango, and passion fruit) [31]. Furthermore, at the family level, *Cladosporiaceae* and *Didymellaceae* were the most abundant in the samples collected from the orchard regardless of the ripe stage, while the samples obtained from the market were abundant in *Sclerotiniaceae, Saccharomytetales,* and *Diaporthaceae* (Figure 3C,D).

**Figure 4:**
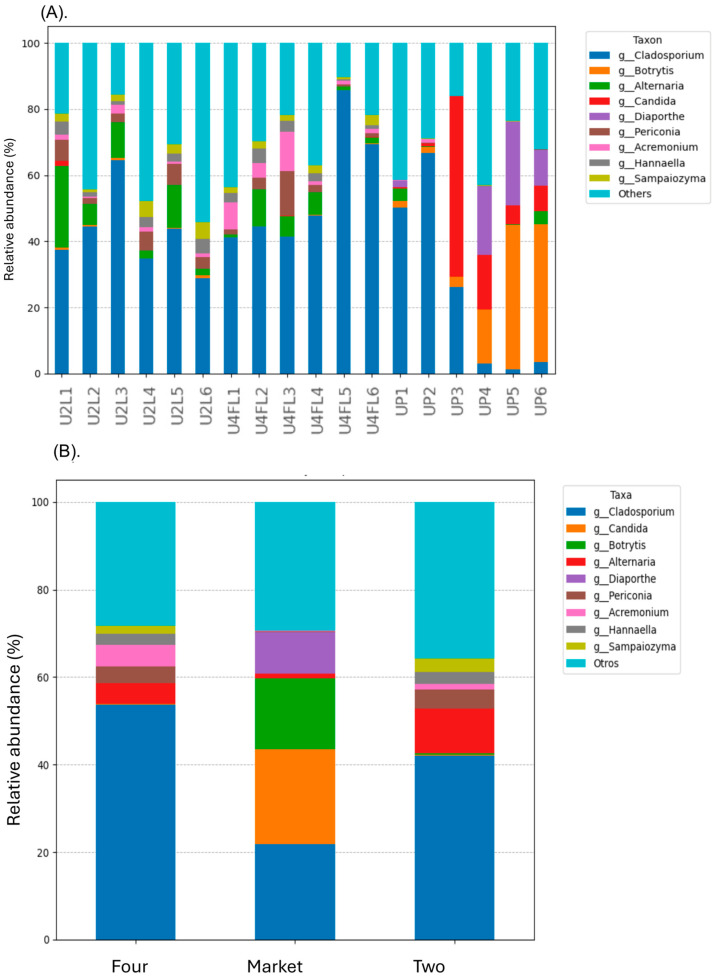
Fungal genera relative abundance (%) across the samples (**A**) and among the groups (**B**). The stacked bar plots were constructed based on the relative abundance of the top 10 fungal genera, while “Other” category was defined as the sum of all classifications with less than 0.50% abundance. Legend: U2L1–U2L6: fruits collected from the organic orchard ripe phase two; U4FL1–U4FL6: fruits collected from the organic orchard ripe phase four; UP1–UP6: fruits purchased from the market sites.

**Figure 5:**
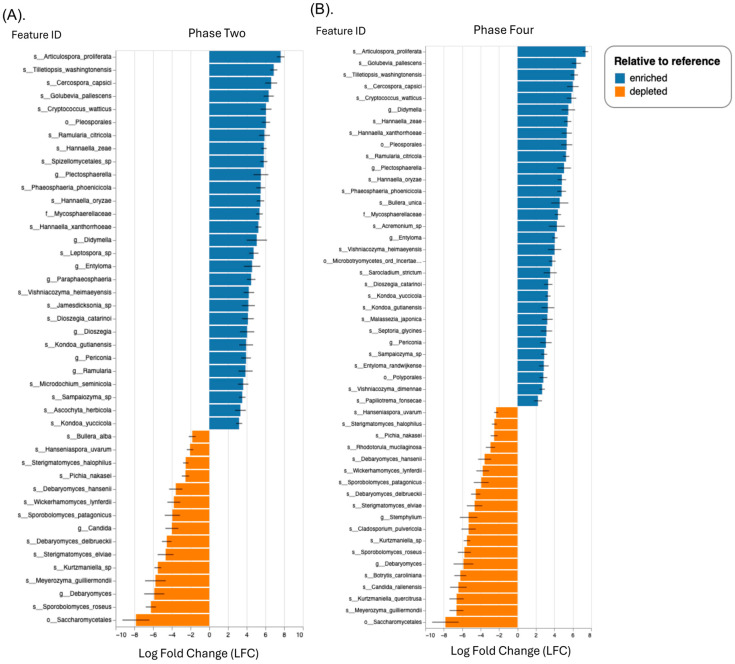
Differential abundance relative to the reference group (market) using ANCOM-BC test. (**A**) Phase two; (**B**) phase four. Y-axis (Feature ID): lists the identifiers of the features (taxa) being compared. X-axis (log fold change, LFC): shows the LFC in abundance of each feature between the groups being compared. A positive value indicates that the feature is more enriched (more abundant) in the group of interest, while a negative value indicates that it is more depleted (less abundant). Error bars: indicate variability or uncertainty in the log fold change estimate. Smaller error bars suggest a more precise estimate.

In the samples obtained from the orchard (phases two and four), *Cladosporium* (relative abundance 41.99–53.77%) and *Alternaria* (relative abundance 4.80–10.06%) were the most prevalent genus levels; in contrast, the samples obtained from the market had relative abundances of 9.57%, 16.32%, and 21.58% for *Candida, Botrytis*, and *Diaporthe*, respectively (Figure 4A,B). Early research on symptomatic *P. peruviana* plants from Pichincha province of Northern Ecuador, showed the presence of *Alternaria, Epicoccum, Diaporthe*, and *Xylaria* [1]. In other studies, *Diaporthe* species have been identified as pathogens responsible for severe canker diseases on plants such as *Vaccinium, Castanea*, *Citrus, Juglans,* and *Pyrus* [32]. In the current study, we confirmed the presence of these genera in the fruit originating from the market and to a lesser extent (<0.001) in the fruits obtained from the orchard. The high content of *Diaporthe* in the market fruits suggests an enhancement of endophyte colonization at the market sites. Furthermore, *Cladosporium* spp. is known as a pathogen that produces malformed fruits and green-gray sporulation on dead tissue [33,34]. In our study, fruits originating from the organic orchard phase four (yellow) showed a high content of the phytopathogen *Sarocladium strictum* (relative abundance of 3.03%). In general, these filamentous fungi are found in soil and plants debris; nonetheless they were proven to be the cause of neurological illness in patients with compromised immune systems when antifungal therapy is ineffective [35]. Likewise, previous research showed that the most significant spoilage microorganisms in cape gooseberries cultivated in Turkey were *Botrytis cinerea* and *Alternaria alternata,* which reduced their shelf life to only 1–2 weeks at room temperature [36]. In the current study, no *B. cinerea* was detected. Instead, the fungal pathogens, *Candida railenensis* with a relative abundance of 21.54%, and *B. caroliniana* with a relative abundance of 16.32% were found in fruits originating from the market. In our previous 16S metagenomic study in strawberries collected from both orchard and market, *B. caroliniana* (gray mold), and *A. alternata* (black leaf spot) colonized healthy strawberries regardless of their origin (orchard or market) [37]. Although we did not observe any obvious deterioration or damage on the fruit surface, the relative abundance of these fungal pathogens was higher in the cape gooseberries originating from the market, suggesting that the market favors the growth of some filamentous fungi or there is a cross-contaminated from the market resident fruits or vegetables. Moreover, based on the differential abundance ANCOM-BC test, we examined which taxa were significantly different in abundance between the conditions (market vs. phase two; market vs. phase four). However, a significant increase in the mean absolute abundance of several beneficial fungal taxa, such as *Articulospora proliferata* (1.30–1.77%) and *Tilletiopsis washingtonenses* (0.52–0.83%)*,* were found in the samples from the orchard regardless of the phase, whereas the market samples showed high abundance of *Sporobolomyces roseus, Debaryomyces* sp., *Meyerozyma guilliermondii,* and *Kurtzmaniella* sp. (Figure 5A,B). In a recent study evaluating the mycobiome on bagged-apple peel among several taxa, *A. proliferata* have been reported to exhibit antifungal activity or growth-promoting activity [12]. Furthermore, a study comparing the ITS and D1/D2 domains of the large subunit of ribosomal RNA gene (LSU rRNA) revealed the presence of nine species within the genus *Tilletiopsis* in a variety of apple cultivars [38]. The growth of these fungi was linked to the phenomenon known as white haze disorder [38]. These yeasts were not reported previously in cape gooseberries thus, we do not know their impact on fruit quality. In the market sample, these taxa were not detected, suggesting that at postharvest stage their growth or colonization is blocked. The plant pathogens *Cercospora capsica* (0.61–0.87%) and *Golubevia pallescens* (1.13–1.46%) were abundant in the samples originating from the orchard. A recent metabarcoding analysis carried out to characterize both epiphytic and endophytic microbial communities of apples revealed that *G. pallescens* was most abundant in the epiphytic communities of “Ambrosia” apples at harvest and postharvest [39]. The ascomycete fungus *Cercospora*, found in tropical and subtropical areas of the world, causes leaf lesions and defoliation on fruit [40]. Given that the cape gooseberries were harvested from an organic orchard, there is a significant chance that microorganisms will contaminate the fruit, with negative impact on its quality and safety after harvest.

### 3.4. Fruits Spoilage Increased at the Market Sites

**Figure 6:**
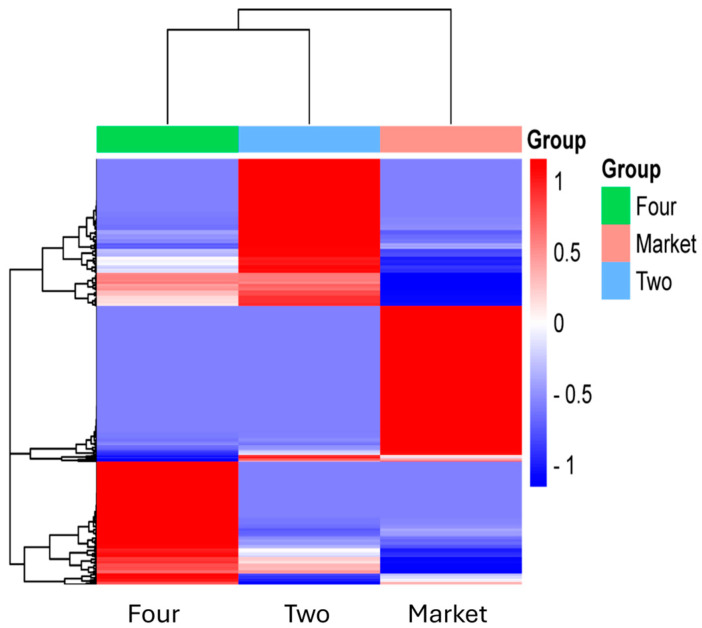
Heatmap and hierarchical clustering of the most abundant fungi at the genus level. On the X-axis are the experimental conditions that are being compared (groups); Y-axis contains the microbial genus that has been identified in the group; blue intense color: low abundance, red color: high abundance.

Tropical fruits are particularly vulnerable to microbial deterioration. While in storage, transit, or processing, fruits can become microbially spoiled [40]. Because of the physiological and compositional changes caused by this spoiling, quality is lost, and shelf life is greatly reduced [41]. Fruit peels’ natural microbiota profiling offers comprehensive information about the variety and relative abundance of various fungal communities colonizing organically produced fruits. A recent study in Colombia showed that the main spoilage culprits causing the cape gooseberries’ deterioration were *Botrytis cinerea* and *Penicillium cyclopium* [8]. In addition, the yeasts *M. guilliermondii* and the bacterium *Pantotea* spp. were detected, and their presence was associated with the cultivation region [8]. In the current study, a clear difference in the mycobiome was observed based on the abundance data of the major genera/species of all comparison groups (Figure 6). Despite not having any visible damage, fruits from the market were found to contain species from the genera *Botrytis* (*B. caroliniana* with a relative abundance of 16.32%) and *Penicillium* (*P. digitatum* with a relative abundance of 1.56%) (Figure S3). The green mold *P. digitatum* is detected in citric fruits and usually grows rapidly at higher temperatures and produces the infection in postharvest fruits [42]. According to Oluwadara and col. [41], there is a possibility that saprophytes can proliferate due to prior plant pathogen-induced harm. On the other hand, fruit cracks can transmit plant phytopathogens, increasing spoiling and waste. Even though the fruits in this study were selected after being transported to the lab and any cracked fruits were removed, the spores may still survive; this might explain the increased content of fungi in the market fruits. In addition, the genera *Kurtzmaniella* and *Meyerozyma* with a relative abundance < 1% were detected in the samples obtained from the market only; their relative abundance varies with the site. We reported these genera in strawberry fruits that were purchased from the same market, implying that the fruit’s surface may have been cross-contaminated [37].

Furthermore, *M. guilliermondii* has been detected in commercial fruits and vegetables from Russia [43]. Our results, however, emphasize the need for additional microbiological crop control measures in addition to the development of novel standards for the identification of yeasts that are clinically significant in fresh produce. Additionally, *Kurtzmaniella quercitrusa* was found in fruits exclusively from the market, with a relative abundance of 0.7%. These yeasts were previously reported in fermented coffee beans in Ecuador; their growth was associated with the increase in sugars [44]. Nonetheless, the impact on the cape gooseberries’ quality was not documented. We also found that the relative abundances of *Debaryomyces prosopidis* and *Fusarium poae* in the market samples were 2.84% and 1.52%, respectively. Although the abundance of both taxa in the phase two (0.29% and 0.83%) and phase four (0.09% and 0.02%) samples were much lower, we propose that certain taxa may grow after fruit harvest if the storage conditions are suitable (high temperature). *D. prosopidis*, a yeast previously unreported in cape gooseberries, was also discovered in strawberries that were bought from the market. Likewise, *F. poae*, is a plant pathogen that was detected in cereals and foods, their presence is associated with mycotoxin contamination [45]. Toxin-laden food and feed generally pose a major risk to the health of both humans and animals [46]. *Diaporthe kochmanii* with a relative abundance of 5.3% was found in the samples UP4 and UP5 of the market group only. Early studies indicated that species of *Diaporthe* genera are associated with stem canker in several plants [46].

This is the first study detecting these pathogens in cape gooseberry fruits, nonetheless their impact on fruit quality must be evaluated. More research is required to determine whether similar fruit mycobiome patterns are maintained in different settings during different times of the year and whether there is any underlying basis for market fruit susceptibility.

## 4. Conclusions

We have characterized the fungal mycobiome of cape gooseberries collected from an organic certified orchard and market for the first time. Hence, in most of the fruit peels from both unripe and ripe stages, a variety of fungal species were discovered. Although the market samples showed a high abundance of harmful yeasts and molds such as *Meyerozyma guilliermondii, Candida railenensis*, and *Botrytis caroliniana,* the orchard samples showed a notable increase in various beneficial fungal taxa, including *Tilletiopsis washingtonenses* and *Articulospora proliferata*, regardless of the phase, raising the concern about the possible increase in epiphyte fungi postharvest. By using PCoA to identify the most notable output of fungal diversity, a distinct separation of a fungal constituent was established between market and orchard. While it is unclear how these microbes impact fruit quality after harvest, understanding the diversity and variation of mycobiome composition in both ripe and unripe stages is crucial to control microbial spoiling postharvest to reduce the negative effects on the environment, human health, and food security and safety. Our results, however, emphasize the need for additional microbiological crop control measures and the development of novel standards for identifying the presence of yeasts and molds in fresh, ready-to-eat foods. Additionally, the relative abundance of *Basidiomycetes* may be further utilized to identify certain species that are important for nutrition and medicine.

## Figures and Tables

**Table 1:** Sequences characteristics and Shannon diversity index.

Samples ID	Origin/Phase	Total Counts	Filtered Counts	Shannon Diversity Index
U2L1	Orchard/Two	303,908	172,552	3.62
U2L2		274,309	163,847	2.64
U2L3		280,980	161,797	2.69
U2L4		265,145	134,630	4.59
U2L5		333,404	212,034	3.84
U2L6		322,224	208,781	5.03
U4FL1	Orchard/Four	294,183	164,353	4.00
U4FL2		263,105	144,269	3.94
U4FL3		265,678	132,929	3.68
U4FL4		271,939	139,378	3.73
U4FL5		254,197	145,195	1.42
U4FL6		162,737	79,870	2.62
UP1	Market/Four	344,719	255,777	3.07
UP2		330,672	241,965	2.37
UP3		863,540	661,719	1.94
UP4		677,501	489,381	3.38
UP5		421,843	306,856	3.02
UP6		404,413	300,106	3.28

Legend: U2L1–U2L6: fruits collected from the organic orchard phase two; U4L1–U4L6: fruits collected from the organic orchard phase four; UP1–UP6: fruits purchased from the market sites.

**Table 2:** Beta-diversity metrics. The significance was determined through 999 Monte Carlo permutations; the values were considered significant when *p* < 0.05.

Group Comparison	Metrics	Pseudo-F	*p*-Value	q-Value
Four vs. Market	Jaccard distance	3.568	0.002	0.005
Four vs. Two		1.181	0.009	0.009
Market vs. Two		3.452	0.003	0.005
Four vs. Market	Bray–Curtis dissimilarity	5.467	0.006	0.009
Four vs. Two		1.254	0.266	0.266
Market vs. Two		5.440	0.003	0.009
Four vs. Market	Unweighted_unifrac distance	4.474	0.004	0.006
Four vs. Two		1.205	0.053	0.053
Market vs. Two		4.202	0.003	0.006
Four vs. Market	Weighted_unifrac distance	8.021	0.006	0.009
Four vs. Two		2.069	0.095	0.095
Market vs. Two		9.897	0.001	0.003

## Data Availability

Raw sequence data were deposited in the National Collection of Biotechnology Information under Bioproject ID PRJNA1158320 on 11 September 2024 (https://www.ncbi.nlm.nih.gov/sra/PRJNA1158320).

## Supplementary Materials

The following supporting information can be downloaded at: https://www.mdpi.com/article/10.3390/foods13203248/s1, Figure S1. Cape gooseberry plants in the orchard at unripe (A) and ripe (B) stages. Collected fruits green—unripe (C) and yellow—ripe (D). Figure S2: Refraction curves illustration. Figure S3: Fungal species (relative abundance %) across the samples (A) and groups (B) identified in cape gooseberry. Table S1. Filtered reads upon employment of DADA2 statistics; Table S2. Alpha-diversity metrics; Table S3. Taxon relative abundance between groups.
