# Supplementary material for: Deciphering the Cape Gooseberry Fruits Mycobiome for Further Safety Improvement Postharvest
Source: Foods. 2024 Oct 12;13(20):3248. doi: 10.3390/foods13203248 (PMC11506962; doi:10.3390/foods13203248)

Supplementary Materials

Table S1. Filtered reads upon employment of DADA2 statistics

Samples ID	Origin/ Phase	Input	Filtered	Percentage of input passed filter	Denoised	Merged	Percentage of input merged	Non-chimeric	Percentage of input non-chimeric
**U2L1**	Orchard/ Two	303908	233424	76.81	233257	220619	72.59	220328	72.5
**U2L2**		274309	215003	78.38	214887	203941	74.35	203838	74.31
**U2L3**		280980	220556	78.5	220182	218851	77.89	218343	77.71
**U2L4**		265145	205746	77.6	205570	204596	77.16	203934	76.91
**U2L5**		333404	259446	77.82	259375	257537	77.24	256674	76.99
**U2L6**		322224	234570	72.8	234441	233040	72.32	232642	72.2
**U4FL1**	Orchards/ Four	294183	220675	75.01	220538	219187	74.51	219057	74.46
**U4FL2**		263105	197631	75.11	197327	196333	74.62	196192	74.57
**U4FL3**		265678	198770	74.82	198563	197808	74.45	197609	74.38
**U4FL4**		271939	207397	76.27	207198	206118	75.8	206002	75.75
**U4FL5**		254197	198217	77.98	198061	196372	77.25	196071	77.13
**U4FL6**		162737	114988	70.66	114910	114106	70.12	114046	70.08
**UP1**	Market	344719	269723	78.24	268945	267011	77.46	266706	77.37
**UP2**		330672	255818	77.36	255373	253237	76.58	252214	76.27
**UP3**		863540	694274	80.4	693209	690190	79.93	667507	77.3
**UP4**		677501	513974	75.86	513357	510270	75.32	498088	73.52
**UP5**		421843	325752	77.22	325035	322332	76.41	310667	73.65
**UP6**		404413	312844	77.36	312587	310960	76.89	308826	76.36

Legend: U2L1-U2L6: fruits collected from the organic orchard phase two; U4L1-U4L6-fruits collected from the organic orchard phase four; UP1-UP6: fruits purchased from market phase four.

Table S2. Alpha-diversity metrics. The significance was determined Kruskal-Wallis; the values were considered significant when $p < 0.05$.

A). Faith

**Group 1**	**Group 2**	**H**	**p-value**	**q-value**
**Four (n=6)**	Market (n=6)	$0.410	$0.522	$0.873
**Four (n=6)**	Two (n=6)	$0.231	$0.631	$0.873
**Market (n=6)**	Two (n=6)	$0.026	$0.873	$0.873

B). Evenness

**Group 1**	**Group 2**	**H**	**p-value**	**q-value**
**Four (n=6)**	Market (n=6)	$1.641	$0.200	$0.262
**Four (n=6)**	Two (n=6)	$1.256	$0.262	$0.262
**Market (n=6)**	Two (n=6)	$2.564	$0.109	$0.262

C). Shannon

**Group 1**	**Group 2**	**H**	**p-value**	**q-value**
**Four (n=6)**	Market (n=6)	$1.641	$0.200	$0.300
**Four (n=6)**	Two (n=6)	$0.410	$0.522	$0.522
**Market (n=6)**	Two (n=6)	$2.564	$0.109	$0.300

D). Observed_features

**Group 1**	**Group 2**	**H**	**p-value**	**q-value**
**Four (n=6)**	Market (n=6)	$0.315	$0.575	$0.631
**Four (n=6)**	Two (n=6)	$0.231	$0.631	$0.631
**Market (n=6)**	Two (n=6)	$0.410	$0.522	$0.631

Table S3. Taxon relative abundance between groups

**Taxon**	**Four**	**Market**	**Two**
p__Ascomycota	86.5511406	96.7679816	85.7234105
p__Basidiomycota	13.3662285	3.21512862	13.9168844
p__Chytridiomycota	0.07072013	0	0.35666797
p__Mucoromycota	0.00955342	0.01587017	0.00142363
p__Mortierellomycota	0.00235734	0.00017732	0
p__Fungi_phy_Incertae_sedis	0	0.00084227	0.00161345

Figure S1. Cape gooseberry plants in the orchard at unripe (A) and ripe (B) stages. Collected fruits green-unripe (C) and yellow-ripe (D).

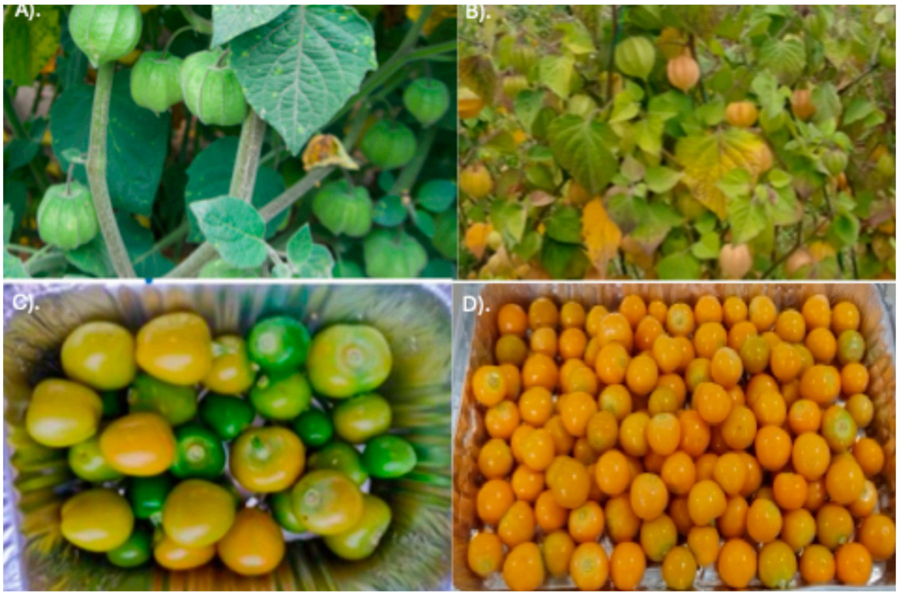

Figure S2. Refraction curves illustration. Legend: U2L1-U2L6: fruits collected from the organic orchard phase two; U4L1-U4L6-fruits collected from the organic orchard phase four; UP1-UP6: fruits purchased from market phase four.

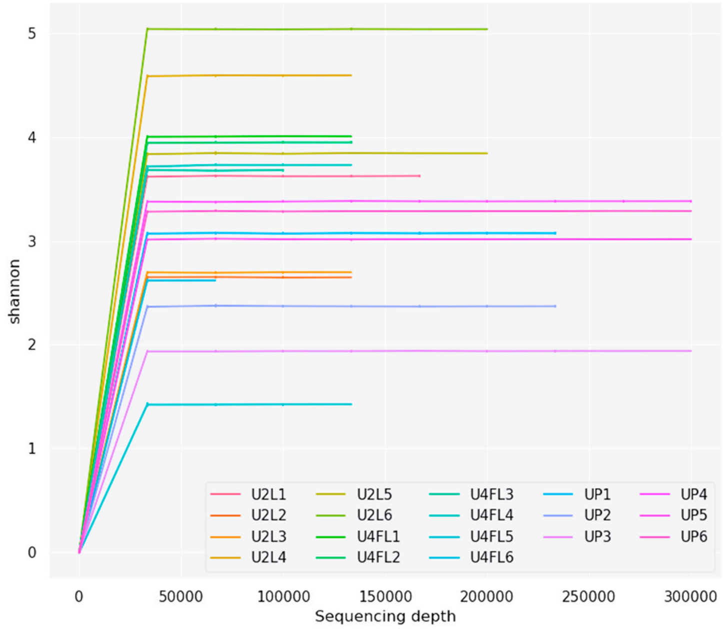

Figure S3. Fungal species (relative abundance %) across the samples (A) and groups (B) identified in cape gooseberry. The stacked bar plots shows were constructed based on the relative abundance of the top 10 fungal genera, while "Other" category was defined as the sum of all classifications with less than 0.50% abundance. Legends: U2L1-U2L6: fruits collected from the organic orchard ripe phase two; U4FL1-U4FL6: fruits collected from the organic orchard ripe phase four; UP1-UP6: fruits purchased from market.

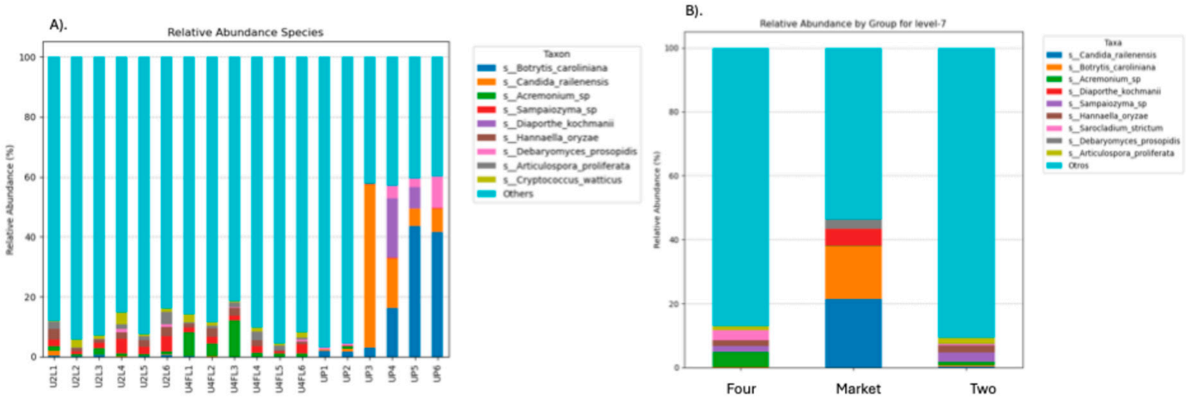

Supplement: Supplementary file 1 [file foods-13-03248-s001.zip › foods-3233902-supplementary.pdf]
